# Diffusion kurtosis imaging can efficiently assess the glioma grade and cellular proliferation

**DOI:** 10.18632/oncotarget.5675

**Published:** 2015-11-03

**Authors:** Rifeng Jiang, Jingjing Jiang, Lingyun Zhao, Jiaxuan Zhang, Shun Zhang, Yihao Yao, Shiqi Yang, Jingjing Shi, Nanxi Shen, Changliang Su, Ju Zhang, Wenzhen Zhu

**Affiliations:** ^1^ Department of Radiology, Tongji Hospital, Tongji Medical College, Huazhong University of Science and Technology, Wuhan, China

**Keywords:** diffusion kurtosis imaging, glioma, grading, cellular proliferation, Ki-67

## Abstract

Conventional diffusion imaging techniques are not sufficiently accurate for evaluating glioma grade and cellular proliferation, which are critical for guiding glioma treatment. Diffusion kurtosis imaging (DKI), an advanced non-Gaussian diffusion imaging technique, has shown potential in grading glioma; however, its applications in this tumor have not been fully elucidated. In this study, DKI and diffusion weighted imaging (DWI) were performed on 74 consecutive patients with histopathologically confirmed glioma. The kurtosis and conventional diffusion metric values of the tumor were semi-automatically obtained. The relationships of these metrics with the glioma grade and Ki-67 expression were evaluated. The diagnostic efficiency of these metrics in grading was further compared. It was demonstrated that compared with the conventional diffusion metrics, the kurtosis metrics were more promising imaging markers in distinguishing high-grade from low-grade gliomas and distinguishing among grade II, III and IV gliomas; the kurtosis metrics also showed great potential in the prediction of Ki-67 expression. To our best knowledge, we are the first to reveal the ability of DKI to assess the cellular proliferation of gliomas, and to employ the semi-automatic method for the accurate measurement of gliomas. These results could have a significant impact on the diagnosis and subsequent therapy of glioma.

## INTRODUCTION

Gliomas are the most common type of intra-axial brain tumors. They have different cellular origins and are divided into four grades according to the World Health Organization (WHO) criteria [1]. Accurate grading is critical for glioma treatment and prognosis. However, patients with the same type of tumor and who receive equivalent treatment doses might display diverse outcomes due to the varying proliferative activities of their tumors [2]. Therefore, accurate prediction of proliferation is also particularly important in gliomas.

Conventional diffusion weighted imaging (DWI) of three orthogonal directions and diffusion tensor imaging (DTI) are not sufficiently accurate in evaluating the glioma grade [3–6] and Ki-67 expression [7, 8], a nuclear antigen expressed in proliferating cells that indicates cellular proliferation. This insufficiency is because standard DWI and DTI assume that water diffusion has a Gaussian distribution. However, due to the complexity of the structure of brain tissue and cells, including cell membranes, intracellular organelles, and water compartments, the diffusion of water molecules tends to deviate from a Gaussian distribution [9], thereby limiting the effectiveness of conventional DWI and DTI.

Diffusion kurtosis imaging (DKI) is an advanced non-Gaussian diffusion imaging technique that can be used to account for this deficiency. It provides a more accurate model of diffusion for quantifying the deviation from a Gaussian distribution, which is known as kurtosis [10]. By acquiring data for at least two nonzero diffusion gradient factors (*b* value) in more than 15 nonlinear directions, the kurtosis metrics (including mean kurtosis (MK), axial kurtosis (Ka) and radial kurtosis (Kr)) and conventional diffusion metrics (including mean diffusivity (MD), axial diffusivity (Da), radial diffusivity (Dr) and fractional anisotropy (FA)) are obtained simultaneously. Ka is parallel to the main direction of diffusion, Kr is perpendicular to the main direction of diffusion, and MK is the average kurtosis of all diffusion directions [11]. Ka might reflect the axonal integrity and density of fiber bundles, and Kr might reflect the myelin integrity and axonal density [9]. As an extension of DTI, DKI can provide additional kurtosis information, which is generally assumed to be caused by tissue microstructure, and it is believed to be generally proportional to the heterogeneity and complexity of the microstructure [12–14]. Additionally, DKI can detect changes in gray matter and fiber crossing [9, 15]. Thus far, DKI has shown utility in ischemia and infarction [16], traumatic brain injury [17], neoplasm [9, 14, 15, 18], neurodegenerative disease [19, 20], and demyelinating diseases [21]. Accordingly, DKI may be more suitable than DWI and DTI for the detection of microstructural changes in tissues and cells.

Gliomas comprise a heterogeneous group of tumors characterized by increased microstructural complexity and heterogeneity, especially for higher grade gliomas or gliomas with higher cellular proliferation, which might impede proton diffusion and lead to higher non-Gaussianity and increased kurtosis [13, 14]. Although DKI has shown potential in grading gliomas [9, 14, 15], the sample size was limited in these studies, and no comparison of DKI metrics with apparent diffusion coefficient (ADC) was reported. Moreover, the relationship between DKI and the proliferative activity of glioma cells was not evaluated. Therefore, the roles of DKI in gliomas still have not been fully elucidated.

In this study, we assessed and compared the value of the kurtosis metrics (MK, Ka and Kr) and conventional diffusion metrics (MD, FA and ADC) in grading gliomas and also evaluated the correlation between these metrics and the Ki-67 labeling index (Ki-67 LI). Our results demonstrate that the kurtosis metrics are more promising imaging markers in grading, and also had great potential in the prediction of cellular proliferation in gliomas.

## RESULTS

### Patient groups

DKI and DWI scans were performed on 102 patients with suspected glioma between July 2012 and June 2014. Of these 102 patients, 23 were excluded because they failed to undergo resection or biopsy, and another 5 were excluded because their lesions were histopathologically confirmed to be non-gliomas. Ultimately, a total of 74 patients were included in this study, and all of them underwent tumor resection. According to WHO criteria, 3 had grade I glioma, 31 had grade II, 19 had grade III, and 21 had grade IV. Other detailed information for the patients is shown in Supplementary Table S1 online. The tumor characteristics from routine MRI are shown in Supplementary Table S2 online. The maximum time between MRI and surgery was 25 days. The most common symptoms were epilepsy, headache, limb weakness, nausea, and vomiting.

### Value of the kurtosis and conventional diffusion metrics in grading gliomas

**Figure 1:**
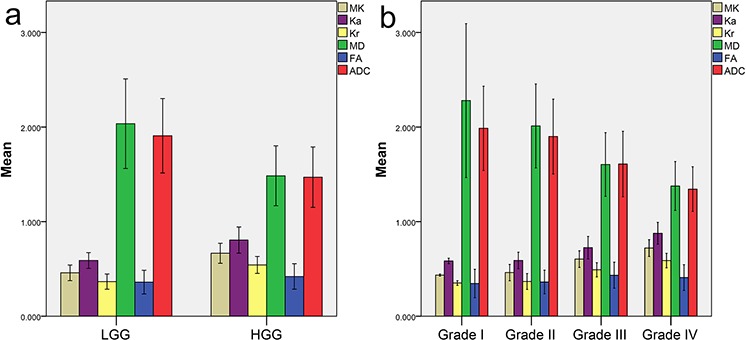
Bar charts of normalized metrics for different grade gliomas Bar chart of each normalized metric **a.** in HGG and LGG and **b.** in grade I, grade II, grade III and grade IV glioma. All metrics are dimensionless, except for MD and ADC, with units of 10^−3^ mm^2^/sec. MK: mean kurtosis; Ka: axial kurtosis; Kr: radial kurtosis; MD: mean diffusivity; FA: fractional anisotropy; ADC: apparent diffusion coefficient; HGG: high-grade glioma; LGG: low-grade glioma.

The kurtosis and conventional diffusion metric values in the solid region of the tumor and contralateral normal-appearing white matter (NAWM) are reported as the mean and standard deviation in Table 1. The inter-observer variability of measurements in 30 randomly selected patients is reported in Table 2. The intra-class correlation coefficients for inter-observer were between 0.673 and 0.980, and the reproducibility between observers was excellent. The metric values in the solid region of the tumor were first normalized to eliminate whole-brain variations between individuals. The corresponding bar charts of the normalized metrics for different grade gliomas are shown in Figure 1. Next, differences in each metric between high-grade glioma (HGG) and low-grade glioma (LGG) were compared using the independent-samples *t*-test. The results demonstrated that the kurtosis metrics were significantly higher in the HGGs compared with the LGGs (MK: 0.665 ± 0.106 vs. 0.459 ± 0.082, Ka: 0.805 ± 0.139 vs. 0.589 ± 0.083, Kr: 0.542 ± 0.089 vs. 0.366 ± 0.080; *P* < 0.001 for all). In contrast, MD and ADC were significantly lower in the HGGs compared with the LGGs (MD: 1.485 ± 0.316 vs. 2.035 ± 0.474; ADC: 1.470 ± 0.319 vs. 1.907 ± 0.394; *P* < 0.001 for both). However, FA did not differ significantly between the two groups (0.420 ± 0.135 vs. 0.360 ± 0.125; *P* = 0.052). Therefore, all metrics except FA were able to well distinguish HGGs from LGGs.

**Table 1:** Kurtosis and conventional diffusion metric values in the solid region of the tumor and the contralateral NAWM

Region	Number	MK	Ka	Kr	MD (10^−3^ mm^2^/sec)	FA	ADC (10^−3^ mm^2^/sec)
Solid region of the tumor							
Overall	74	0.586 ± 0.140	0.569 ± 0.135	0.605 ± 0.147	1.466 ± 0.410	0.163 ± 0.054	1.228 ± 0.303
LGGs	34	0.484 ± 0.088	0.472 ± 0.078	0.500 ± 0.102	1.720 ± 0.412	0.154 ± 0.050	1.404 ± 0.293
HGGs	40	0.674 ± 0.113	0.651 ± 0.118	0.695 ± 0.118	1.251 ± 0.259	0.170 ± 0.057	1.078 ± 0.220
Grade I gliomas	3	0.427 ± 0.022	0.408 ± 0.047	0.447 ± 0.044	2.071 ± 0.790	0.156 ± 0.060	1.529 ± 0.486
Grade II gliomas	31	0.489 ± 0.091	0.478 ± 0.078	0.505 ± 0.105	1.686 ± 0.362	0.154 ± 0.051	1.392 ± 0.277
Grade III gliomas	19	0.610 ± 0.095	0.585 ± 0.106	0.632 ± 0.098	1.349 ± 0.282	0.177 ± 0.063	1.178 ± 0.245
Grade IV gliomas	21	0.732 ± 0.097	0.711 ± 0.096	0.751 ± 0.107	1.162 ± 0.203	0.163 ± 0.052	0.987 ± 0.148
Contralateral NAWM	74	1.033 ± 0.058	0.806 ± 0.049	1.327 ± 0.114	0.844 ± 0.035	0.418 ± 0.037	0.737 ± 0.045

Note: Data are presented as the mean and standard deviation. All metrics are dimensionless, except for MD and ADC.

MK: mean kurtosis; Ka: axial kurtosis; Kr: radial kurtosis; MD: mean diffusivity; FA: fractional anisotropy; ADC: apparent diffusion coefficient; HGGs: high-grade gliomas; LGGs: low-grade gliomas; NAWM: normal-appearing white matter.

**Table 2:** Inter-observer variability in measurements of 30 randomly selected patients

Region	Metrics	Intra-class correlation coefficient, 95% CI for Inter-observer
Solid region of the tumor	MK	0.903, 0.796–0.954
	Ka	0.903, 0.796–0.954
	Kr	0.889, 0.768–0.947
	MD	0.871, 0.730–0.939
	FA	0.673, 0.314–0.845
	ADC	0.906, 0.803–0.955
NAWM	MK	0.923, 0.838–0.963
	Ka	0.980, 0.958–0.991
	Kr	0.920, 0.832–0.962
	MD	0.962, 0.920–0.982
	FA	0.902, 0.795–0.954
	ADC	0.972, 0.941–0.987

CI: confidence interval; MK: mean kurtosis; Ka: axial kurtosis; Kr: radial kurtosis; MD: mean diffusivity; FA: fractional anisotropy; ADC: apparent diffusion coefficient; NAWM: normal-appearing white matter.

**Figure 2:**
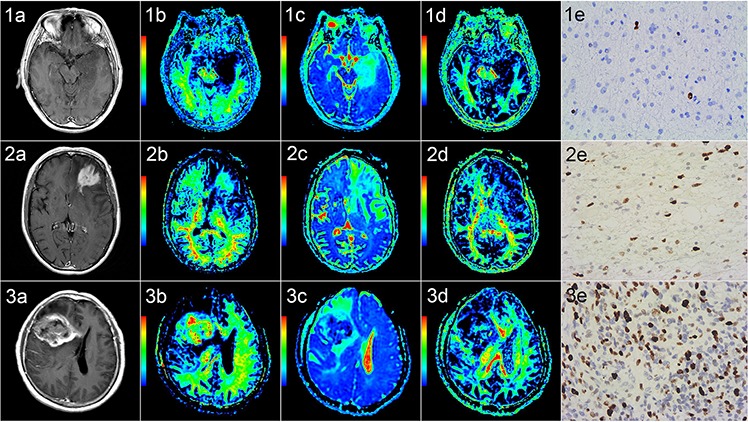
Correlation of diffusion kurtosis imaging with tumor grade and Ki-67 Rows 1–3 correspond to three patients with diffuse astrocytoma (WHO grade II) in the left temporal lobe, anaplastic astrocytoma (WHO grade III) in the left frontal lobe and glioblastoma (WHO grade IV) in the right fronto-temporal lobe, respectively. Columns a-e are contrast-enhanced T1-FLAIR, MK, MD, FA and Ki-67 images (400 ×), respectively. For grade II gliomas, the intensity was low on MK and FA maps and high on MD maps, and the Ki-67 LI value was 2%. For grade III and grade IV gliomas, the intensity was high on MK maps and low on FA and MD maps, and the Ki-67 LI values were 30% and 45%, respectively. MK and Ki-67 increased (but MD decreased) as the grade increased, whereas FA showed no obvious trend. T1-FLAIR: T1 fluid-attenuated inversion recovery; MK: mean kurtosis; MD: mean diffusivity; FA: fractional anisotropy; Ki-67 LI: Ki-67 labeling index.

In addition to differentiating between HGGs and LGGs, the differences among grade II, III and IV gliomas were further compared using one-way ANOVA, and Student-Newman-Keuls tests were used for the multiple comparisons. The kurtosis metrics were significantly different among grade II, III and IV gliomas (MK: 0.461 ± 0.086 for grade II, 0.604 ± 0.088 for grade III, 0.721 ± 0.089 for grade IV, *P* < 0.001; Ka: 0.589 ± 0.087 for grade II, 0.725 ± 0.119 for grade III, 0.877 ± 0.115 for grade IV, *P* < 0.001; Kr: 0.367 ± 0.084 for grade II, 0.491 ± 0.075 for grade III, 0.588 ± 0.077 for grade IV; *P* < 0.001); the differences between each pair of grades were also significant in multiple comparisons (*P* < 0.05). This observation was also the case for MD and ADC among grade II, III and IV gliomas (MD: 2.012 ± 0.443 for grade II, 1.604 ± 0.337 for grade III, 1.376 ± 0.259 for grade IV, *P* < 0.001; ADC: 1.900 ± 0.396 for grade II, 1.609 ± 0.346 for grade III, 1.344 ± 0.236 for grade IV, *P* < 0.001) as well as between each pair of grades in multiple comparisons (*P* < 0.05). However, FA did not show significant differences among grade II, III and IV gliomas (0.362 ± 0.125 for grade II, 0.433 ± 0.137 for grade III, 0.409 ± 0.136 for grade IV, *P* = 0.155). As the tumor grade increased, the kurtosis metrics showed an increasing trend; in contrast, a decreasing trend was demonstrated for MD and ADC, whereas no obvious trends were found for FA, as shown in Figure 2. Therefore, all metrics except FA were able to satisfactorily identify glioma grades II to IV.

### Comparisons of the diagnostic efficiency of the kurtosis and conventional diffusion metrics in differentiating tumor grades

**Figure 3:**
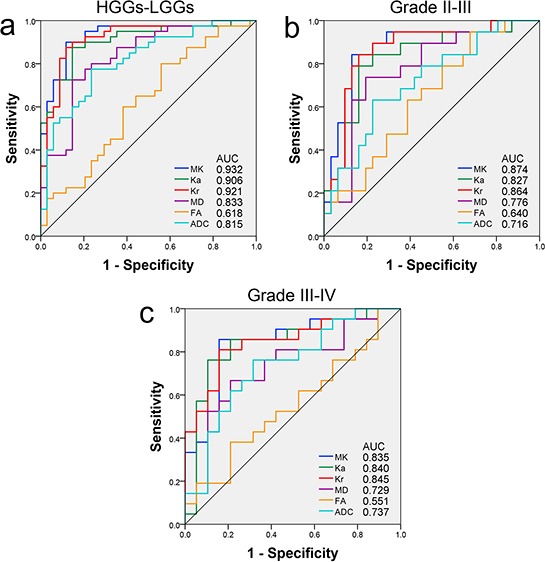
ROC curves for all the metrics in differentiating tumor grades ROC curves and AUCs for all the metrics in the solid region of the tumor for the differentiation **a.** between HGGs and LGGs, **b.** between grade II and III and **c.** between grade III and IV gliomas. ROC curves: receiver operating characteristic curves; AUC: area under the curve; MK: mean kurtosis; Ka: axial kurtosis; Kr: radial kurtosis; MD: mean diffusivity; FA: fractional anisotropy; ADC: apparent diffusion coefficient; HGGs: high-grade gliomas; LGGs: low-grade gliomas.

To find the best diagnostic factors for glioma grading, the diagnostic efficiency of each metric was compared using receiver operating characteristic (ROC) curves. The area under the curve (AUC), optimal cut-off value, and corresponding sensitivity and specificity for all metrics used to differentiate between LGGs and HGGs, between grade II and III gliomas, and between grade III and IV gliomas are reported in Table 3; the corresponding ROC curves are shown in Figure 3. The kurtosis metrics exhibited the maximal AUCs and optimal sensitivity and specificity for distinguishing between HGGs and LGGs, grade II and III gliomas and grade III and IV gliomas. MD and ADC had lower ones, followed by FA. The differences in the diagnostic efficiency among the kurtosis metrics were slight, though MK displayed the optimal sensitivity and specificity in all comparisons.

**Table 3:** Statistical values of all metrics for differentiating between HGGs and LGGs, grade II and grade III, and grade III and grade IV gliomas

Metrics	AUC	*P* Value	Cut-off value	Sensitivity	Specificity
HGGs-LGGs					
MK	0.932	< 0.001*	0.553	90.00%	88.20%
Ka	0.906	< 0.001*	0.655	87.50%	85.30%
Kr	0.921	< 0.001*	0.443	87.50%	88.20%
MD(10^−3^ mm^2^/sec)	0.833	< 0.001*	1.573	72.50%	85.30%
FA	0.618	0.081	0.324	80.00%	44.10%
ADC(10^−3^ mm^2^/sec)	0.815	< 0.001*	1.627	77.50%	76.50%
Grade II–III					
MK	0.874	< 0.001*	0.553	84.20%	87.10%
Ka	0.827	< 0.001*	0.655	78.90%	83.90%
Kr	0.864	< 0.001*	0.436	84.20%	83.90%
MD(10^−3^ mm^2^/sec)	0.776	< 0.001*	1.627	73.70%	80.60%
FA	0.640	0.079	0.287	94.70%	32.30%
ADC(10^−3^ mm^2^/sec)	0.716	0.004	1.575	63.20%	77.40%
Grade III–IV					
MK	0.835	< 0.001*	0.667	85.70%	84.20%
Ka	0.840	< 0.001*	0.825	76.20%	89.50%
Kr	0.845	< 0.001*	0.538	81.00%	84.20%
MD(10^−3^ mm^2^/sec)	0.729	0.013*	1.409	66.70%	78.90%
FA	0.551	0.579	0.334	38.10%	78.90%
ADC(10^−3^ mm^2^/sec)	0.737	0.003*	1.400	76.20%	68.40%

Note: The cut-off value indicates the optimal threshold in the current study/sample size. All metrics are dimensionless, except for MD and ADC.

* *P* < 0.05.

AUC: area under the curve; HGGs: high-grade gliomas; LGGs: low-grade gliomas; MK: mean kurtosis; Ka: axial kurtosis; Kr: radial kurtosis; MD: mean diffusivity; FA: fractional anisotropy; ADC: apparent diffusion coefficient.

The AUCs of MK, MD, ADC and FA were further compared because these four metrics are the representative metrics. The results shown in Table 4 demonstrate that the AUC of MK was significantly higher than that of MD in differentiating between LGGs and HGGs, significantly higher than that of ADC in differentiating between LGGs and HGGs and between grade II and III gliomas, and also significantly higher than that of FA in all the differentiations; the AUCs of MD and ADC were significantly higher than that of FA in differentiating between LGGs and HGGs (*P* < 0.05 for all).

**Table 4:** Comparisons of AUCs among MK, MD, ADC and FA

Comparison	Statistic	MK-MD	MK-ADC	MK-FA	MD-ADC	MD-FA	ADC-FA
LGG-HGG	P	0.011*	0.001*	< 0.001*	0.450	< 0.001*	0.001*
	Z	2.537	3.195	4.839	0.755	3.577	3.227
Grades II–III	P	0.077	0.007*	0.003*	0.108	0.111	0.353
	Z	1.770	2.683	3.017	1.608	1.594	0.928
Grades III–IV	P	0.144	0.150	0.020*	0.836	0.178	0.156
	Z	1.462	1.438	2.324	0.207	1.346	1.419

Note:

* *P* < 0.05.

MK: mean kurtosis; MD: mean diffusivity; ADC: apparent diffusion coefficient; FA: fractional anisotropy; HGG: high-grade glioma; LGG: low-grade glioma.

Furthermore, a stepwise multiple logistic regression analysis of MK, MD, ADC and FA was performed to find the most significant metric for the differentiations, and the results demonstrated that MK was a significant predictor positively associated with the glioma grade in the differentiations between HGGs and LGGs (when MK increases by 0.1, odds ratio = 7.291, 95% confidence interval = 3.189–16.667; *P* < 0.001), between grade II and III gliomas (when MK increases by 0.1, odds ratio = 5.423, 95% confidence interval = 2.204–13.344; *P* < 0.001), and between grade III and IV gliomas (when MK increases by 0.1, odds ratio = 4.939, 95% confidence interval = 1.734–14.067; *P* < 0.001); whereas the other metrics were not included in the stepwise multiple logistic regression model in all differentiations.

These data indicated that the kurtosis metrics were superior to conventional diffusion metrics for gliomas grading. The kurtosis metrics could achieve more accurate grading, which may help guide the subsequent treatment for glioma patients.

### Correlation between Ki-67 and the kurtosis and conventional diffusion metrics

The gliomas from 66 patients were subjected to additional immunohistochemistry examination to detect Ki-67 expression. The Ki-67 LI for different grade gliomas are reported as the mean and standard deviation in Supplementary Table S3 online. The difference in Ki-67 LI between HGGs and LGGs was significant (*P* < 0.001). The difference in Ki-67 LI among grade II, III and IV gliomas was also significant (*P* < 0.001), which was also the case for Ki-67 LI between each pair of grades (*P* < 0.05). The Ki-67 LI was higher for higher grade gliomas.

**Figure 4:**
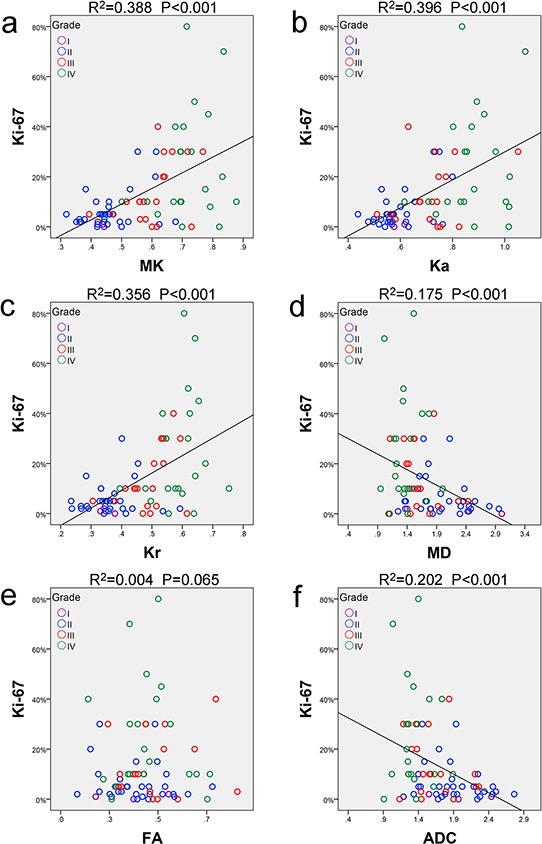
Correlations between Ki-67 and each metric Scatter diagrams demonstrating the correlations between Ki-67 labeling index and **a.** MK, **b.** Ka, **c.** Kr, **d.** MD, **e.** FA or **f.** ADC. All metrics are dimensionless, except for MD and ADC, with units of 10^−3^ mm^2^/sec. MK: mean kurtosis; Ka: axial kurtosis; Kr: radial kurtosis; MD: mean diffusivity; FA: fractional anisotropy; ADC: apparent diffusion coefficient.

As the prediction of cellular proliferation is valuable in the evaluation of tumor behavior, response to therapy and prognosis, the correlations between Ki-67 LI and each metric were evaluated using Pearson correlation analysis. Significant correlations were found between Ki-67 LI and the kurtosis metrics (MK: *r* = 0.623, *P* < 0.001; Ka: *r* = 0.629, *P* < 0.001; Kr: *r* = 0.597, *P* < 0.001), as well as for MD (*r* = −0.418, *P* < 0.001) and ADC (*r* = −0.449, *P* < 0.001). In contrast, FA had no obvious correlation with Ki-67 LI (*r* = 0.065, *P* = 0.603). Corresponding scatter diagrams are shown in Figure 4. The correlation coefficient was maximal for the kurtosis metrics, followed by ADC and MD, and it was minimal for FA.

These results indicated differences in the cellular proliferation levels of different grade gliomas, and this proliferation could be noninvasively predicted using the kurtosis metrics, MD and ADC. The kurtosis metrics offer great potential for providing additional information on the cellular proliferation of gliomas.

### Subgroup analysis of astrocytic tumors

From the 74 glioma patients, 66 with astrocytic tumors were selected for subgroup analysis, including 3 with pilocytic astrocytoma (grade I), 25 with diffuse astrocytoma (grade II), 16 with anaplastic astrocytoma (grade III), 1 with gliomatosis cerebri (grade III) and 21 with glioblastoma (grade IV). The results obtained from subgroup analysis were basically consistent with those of the whole glioma group analysis, and these results are shown in Supplementary Tables S4 to Tables S8 and Supplementary Figures S1 to Figures S3 online.

The kurtosis metrics were significantly higher (but MD and ADC were significantly lower) in the HGGs compared with the LGGs (*P* < 0.001 for all). The kurtosis metrics, MD and ADC were significantly different among grade II, III and IV gliomas (*P* < 0.001 for all), and the differences between each pair of grades were also significant in multiple comparisons (*P* < 0.05 for all) with the exception of MD in the differentiation between grade III and IV astrocytic tumors. The kurtosis metrics increased but MD and ADC decreased as the tumor grade increased. However, FA did not show significant differences between each pair of glioma grades (*P* > 0.05 for all).

Of all the ROC curves, the ROC of the kurtosis metrics exhibited the maximal AUCs and optimal sensitivity and specificity in all differentiations. MD and ADC showed lower ones, followed by FA. The AUC of MK was significantly higher than that of MD, ADC and FA in differentiating between LGGs and HGGs as well as between grade II and III gliomas; the AUCs of MD and ADC were significantly higher than that of FA in differentiating between LGGs and HGGs (*P* < 0.05 for all). Moreover, the results of the stepwise multiple logistic regression analysis of MK, MD, ADC and FA also demonstrated that MK was a significant predictor positively associated with the glioma grade in all differentiations (*P* < 0.001 for all), whereas the other metrics were not included in the stepwise multiple logistic regression model.

Additional immunohistochemistry of Ki-67 was performed for the gliomas from 58 patients, and significant correlations were found between Ki-67 LI and each kurtosis metric, MD or ADC (*P* < 0.001 for all). In contrast, FA had no obvious correlation with Ki-67 LI (*P* > 0.05).

## DISCUSSION

DKI, an advanced non-Gaussian diffusion imaging technique, was used to better evaluate glioma grade and cellular proliferation in the current study because this approach offers distinct advantages. For example, DKI can provide additional information regarding kurtosis, which is generally proportional to the heterogeneity and complexity of the microstructure [14], and the DKI model results in a more accurate quantification of conventional DTI metrics compared with the DTI model [22]. In addition, DKI is clinically feasible because its acquisition time is acceptable in the clinic.

In the present study, the differences in the kurtosis and conventional diffusion metrics between each pair of glioma grades were first compared, and the corresponding diagnostic efficiency of each metric was determined by subsequent ROC curves. The kurtosis metrics in the solid region of the tumor could effectively distinguish between HGGs and LGGs, grade II and grade III as well as grade III and grade IV gliomas, and these metrics showed the highest AUCs and optimal sensitivity and specificity in all the differentiations. MD and ADC also could distinguish between each pair of glioma grades (with the exception of MD in the differentiation between grade III and IV astrocytic tumors), but their AUCs, sensitivity and specificity were lower than those of the kurtosis metrics. These findings are basically in agreement with those of previous researchers except for the results of MD, because previous researchers found that MD could not be used to distinguish between grade II and III gliomas [9, 15]. These comparisons indicate that the kurtosis metrics may be more consistently effective metrics for grading gliomas, whereas MD is not. In this study, the kurtosis metrics increased (but MD and ADC decreased) as the grade increased because higher grade gliomas are characterized by higher cellularity, more nuclear atypia, higher pleomorphism and heterogeneity with vascular hyperplasia, necrosis, hemorrhage, and endothelial proliferation. In contrast, lower grade gliomas consist of more homogeneous nests of well-differentiated cells with lower cell density and larger cells; lower grade gliomas also contain fewer diffusion barriers. Therefore, higher grade gliomas contain greater structural complexity and heterogeneity compared with lower grade gliomas [9, 15, 23], which increases the kurtosis but decreases the diffusion range in higher grade gliomas.

By comparing the AUCs of ROC curves of the kurtosis and conventional diffusion metrics combined with the stepwise multiple logistic regression analysis of MK, MD, ADC and FA, the kurtosis metrics were shown to be the most promising imaging markers for grading gliomas in the present study. These occurred because a series of changes caused by higher grade gliomas greatly increased the heterogeneity and complexity of the microstructure of tissues and cells [9, 15, 23]. Kurtosis is more sensitive and accurate for the detection of microstructural changes [14], and hence, these changes can be detected by the kurtosis metrics at the early stages but are not sufficiently evident enough for MD or ADC to recognize them. Perhaps this is the reason why the kurtosis metrics are more consistently effective metrics in grading gliomas, whereas MD is not consistently helpful in distinguishing between grade II and III or between grade III and IV gliomas. In short, the kurtosis metrics have an advantage over conventional diffusion metrics and offer greater potential as imaging markers in grading gliomas.

In addition, our study assessed the correlations between these metrics and the cellular proliferation of glioma. Ki-67 was chosen as a proxy for evaluating cellular proliferation because Ki-67 detection can be performed easily and routinely. Moreover, Ki-67 detection is considered one of the most reliable methods for evaluating cellular proliferation and for providing information on tumor behavior and on response to treatment and prognosis [2]. In the current study, significant correlations were revealed between Ki-67 LI and the kurtosis metrics, MD and ADC. Increased Ki-67 expression indicates enhanced cell proliferation and mitosis. During interphase of the cell cycle, more proteins, RNA, DNA and other biological macromolecules are synthesized, and tumor cells grow by producing chromosomes, proteins and cytoplasmic organelles [24]. During mitosis, cells round up to a near-spherical shape, and the chromosomes condense and attach to spindle fibers that pull one copy of each chromosome to opposite sides of the cell [25, 26]. In the tumor tissue, higher cell numbers, denser tumors, narrower intercellular space, enlarged nuclei, a high nucleoplasmic ratio and neo-angiogenesis appear [27, 28]. In addition, higher Ki-67 expression generally correlates with higher grade glioma, which is more heterogeneous due to the cellularity, necrosis, neoangiogenesis, hemorrhage, and endothelial proliferation of the tumor [9, 14, 15]. All these changes may increase the heterogeneity and complexity of the microstructure in the tumor and inhibit water molecule movement both inside and outside tumor cells. Because the deviation from Gaussian diffusion is generally assumed to be caused by the tissue microstructure, and kurtosis is believed to be generally proportional to the heterogeneity and complexity of the microstructure [12–14], kurtosis is likely to increase and diffusion range is likely to decrease. Therefore, cellular proliferation of glioma can be noninvasively evaluated using DKI. The kurtosis metrics offer great potential for providing additional information on the cellular proliferation of gliomas.

In contrast, FA was decreased at all grades and also all levels of Ki-67, perhaps because diffusion is restricted to a similar degree in all directions in the solid region of the tumor. A different tumor grade or Ki-67 level only changes the sphere size of the diffusion range, but does not change its shape. In short, FA is of low value in evaluating both glioma grade or cellular proliferation.

Investigating the relationship between glioma grade and DKI measurements has been covered in multiple previous works [9, 14, 15]. However, unlike previous studies, the sample size was relatively larger in this study; thus, in addition to a comparison between LGG and HGG, comparisons between grade II and III gliomas and between grade III and IV gliomas could also be well performed. Additionally, a subgroup analysis of astrocytic tumors was performed to make the results more generalized. Conversely, the differences between subtypes, such as the difference between astrocytic tumors and oligodendrocyte tumors reported by Tietze et al [14], were not evaluated in this study because of the limited number of oligodendrocyte tumor cases. The diagnostic efficiency of each metric was further compared in the present study, and it was demonstrated that the kurtosis metrics were the most promising imaging markers for grading gliomas. Moreover, this study further assessed the correlation between each metric and cellular proliferation in glioma. To our best knowledge, this is the first time that DKI was used to evaluate cellular proliferation of glioma, and the results demonstrated that the kurtosis metrics had great potential in the prediction of cellular proliferation of gliomas.

In acquiring DKI data, we used 2 *b* values in 25 nonlinear directions rather than 5 and 3 *b* values in more than 25 nonlinear directions, as reported in previous studies [9, 15]. This is a standard DKI scan method for the brain, and it is similar to the method suggested by Jensen et al [29]. This method can provide images with sufficient quality, and acquisition can be completed within a relatively short time, which would produce better outcomes in a clinical setting. Meanwhile, a semi-automated method based on threshold segmentation was introduced for delineating the solid region of the tumor. This method was more accurate (especially in delineating small or thin enhanced tumors), objective, and reproducible and was often easier or faster than manual delineation due to the flexibility of ImageJ and its powerful region of interest (ROI) manager. This semi-automated method is similar to the methods widely used in brain tumors [30–32], acute ischemic stroke [32, 33] and brain anatomical structures [34], and most of the studies have demonstrated that these methods are valid for brain application. To our best knowledge, this is the first time that this semi-automatic method was used in glioma measurement. In addition, studies [9, 35] have reported that MK decreases in the frontal aspects of the brain due to aging in elderly humans, and metric values also change significantly with age in NAWM, including MK, Kr, MD and FA. Therefore, we normalized the DKI and DWI metric values in the solid region of the tumor, and used only normalized metrics in this study because they can be used to eliminate whole-brain interindividual variations, and better results were reported for normalized metrics in previous studies [9, 15]. These are the uniqueness of this study.

In conclusion, normalized kurtosis metrics in the solid region of the tumors were better diagnostic factors in distinguishing HGGs from LGGs and identifying grade II, III and IV gliomas; the kurtosis metrics also offered great potential to noninvasively predict the cellular proliferation of gliomas in the studied cohort of patients. Compared with conventional diffusion metrics, the kurtosis metrics show greater potential as imaging markers for the accurate demonstration of the microstructural changes caused by increasing glioma grade and cellular proliferation, which might allow more accurate diagnosis and optimal therapy for glioma patients.

## MATERIALS AND METHODS

### Inclusion and exclusion criteria for patients

This prospective study was approved by the Ethics Committee of Tongji Hospital of Huazhong University of Science and Technology and abided by the statement of ethical standards. Informed consent was obtained from every patient prior to inclusion in our study. The following inclusion criteria were applied in this study: 1) patients who were suspected of having conditions of a) primary cerebral glioma and b) recurrent gliomas untreated for more than 6 months prior to MRI based on conventional radiologic findings; 2) patients who underwent routine MRI, DWI and DKI in the same scanner; and 3) patients with tumors that were histopathologically confirmed as cerebral gliomas by subsequent resection or biopsy. The following exclusion criteria were applied: 1) patients who rejected surgery or underwent surgery more than 4 weeks after DKI; and 2) patients with motion artifacts.

### Data acquisition

All patients underwent magnetic resonance imaging (MRI) prior to surgery with a 3.0T GE MR 750 system (GE Healthcare, Waukesha, WI).

DKI used a spin-echo echo-planar imaging (SE-EPI) diffusion sequence for image acquisition (TR, 6,500 ms; TE, 85 ms; NEX, 1; matrix, 128 × 128; number of sections, 43; sections thickness, 3 mm; spacing, 0 mm; and FOV, 256 × 256 cm^2^). Two images of b0 were acquired, and *b* values of 1,250 and 2,500 s/mm^2^ were applied in 25 uniformly distributed directions. The acquisition time was 5 minutes 45 seconds.

DWI images were also acquired using a SE-EPI sequence (TR, 3,000 ms; TE, 70 ms; NEX, 4; matrix, 160 × 160; number of sections, 20; sections thickness, 5 mm; spacing, 1.5 mm; and FOV, 240 × 240 cm^2^). Images with and without 3 orthogonal directional motion-probing gradients (*b* = 1,000s/mm^2^) were obtained simultaneously. The acquisition time was 42 seconds.

All patients underwent routine and contrast-enhanced MRI. All images served as an anatomic reference for DKI and DWI. The routine MR scans included the following sequences: transverse T1 fluid-attenuated inversion recovery (T1-FLAIR), transverse T2 fast spin echo (T2-FSE) and transverse T2 fluid-attenuated inversion recovery (T2-FLAIR). The following acquisition parameters were applied: TR = 2,992 ms, TE = 24 ms, TI = 869 ms, NEX = 1, matrix = 320 × 320 for T1-FLAIR; TR = 4,599 ms; TE = 102 ms; NEX = 2; matrix = 320 × 224 for T2-FSE; TR = 8,000 ms; TE = 160 ms; TI = 2,100 ms; NEX = 1; matrix = 256 × 256 for T2-FLAIR. The section thickness, spacing, section number and FOV of all routine sequences were 5 mm, 1.5 mm, 20 and 240 × 240 cm^2^, respectively.

All the sequences had the same scan coverage. The scan plane paralleled the line combining anterior and posterior commissure, and the range covered the entire brain.

### Data processing and analysis

The DKI data were processed using Diffusional Kurtosis Estimator (version 2.5.1, Medical University of South Carolina), and the DWI images were processed using the image calculator of ImageJ (Version 1.49b, NIH). Metric maps were calculated, including MK, Ka, Kr, MD, FA and ADC.

Before delineating the ROI, the image resolution, number of slices and FOV of enhanced T1-FLAIR, T2-FSE and ADC maps should be changed to match the DKI metric maps. Although the scan matrix, slice thickness, spacing and FOV were different in enhanced T1-FLAIR, T2-FSE, DWI and DKI, the image resolutions ultimately generated by the scanner was 512 × 512, 512 × 512, 256 × 256 and 256 × 256, respectively, due to interpolation; in addition, these four sequences had the same scan coverage. Thus, the image resolution of enhanced T1-FLAIR, T2-FSE and ADC was first resized to 240 × 240 (pixels were resized to 1 mm × 1 mm to match the DKI metric maps); the number of slices was changed to 43 without interpolation; and the canvas size was adjusted to 256 × 256 due to the difference in FOV. All these protocols were finished in ImageJ, as shown in Supplementary Figure S4 online.

**Figure 5:**
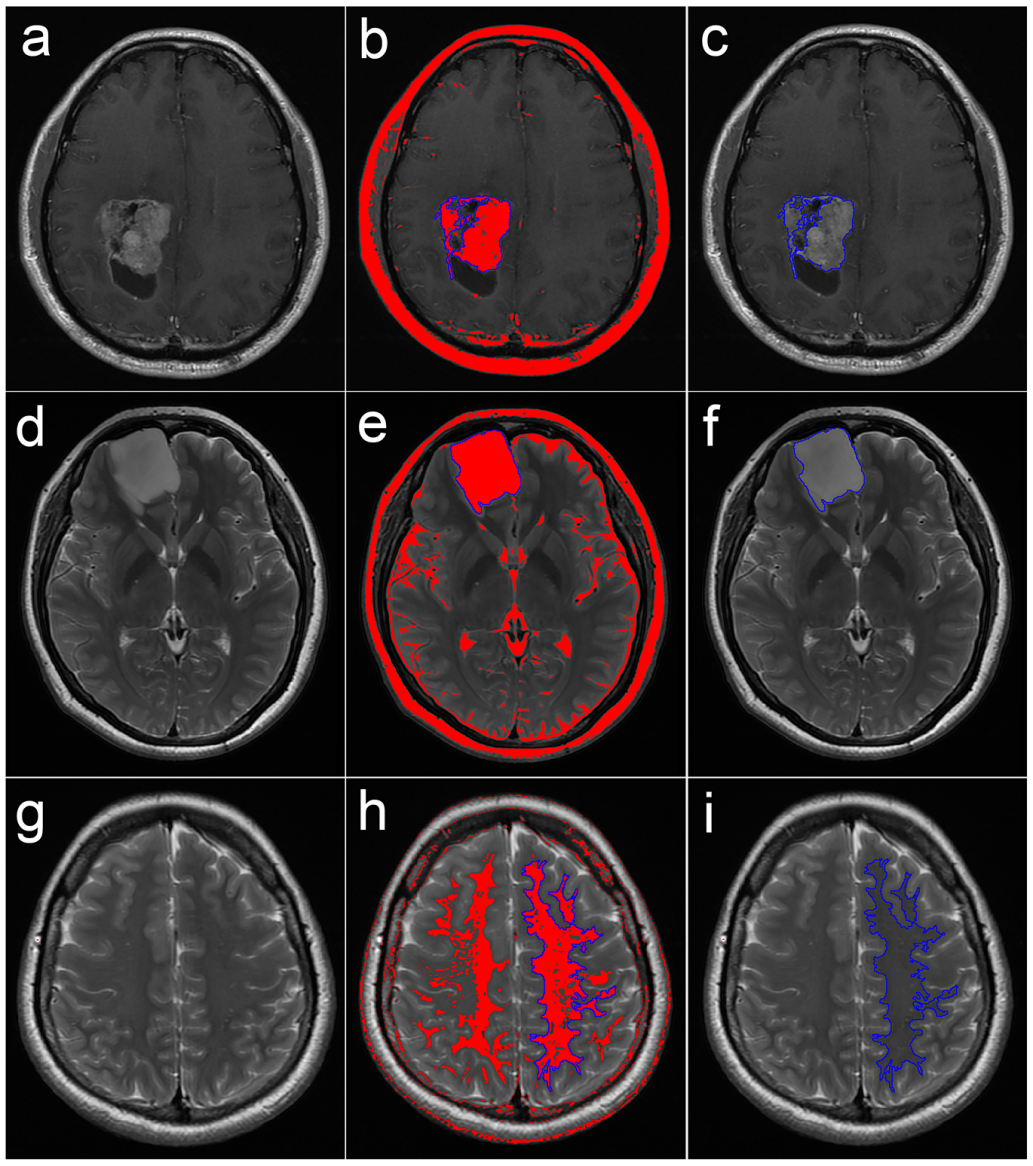
Semi-automated delineation of the ROIs over the solid region of the tumor and the NAWM **a-c.** Delineation over a solid enhancing tumor on transverse contrast-enhanced T1-FLAIR; **d-f.** delineation over a non-enhancing tumor on transverse T2-FSE; and **g-i.** delineation over a contralateral NAWM on transverse T2-FSE. When a proper threshold range of signal intensity was set, the corresponding pixels in the range were colored red, and then the wand tool was used to automatically delineate the connective pixels as the ROI (in blue). ROI: region of interest; NAWM: normal-appearing white matter; T1-FLAIR: T1 fluid-attenuated inversion recovery; T2-FSE: T2 fast spin echo.

ROIs over the solid region of the tumor and NAWM were semi-automatically delineated using the wand tool in ImageJ by an experienced neuroradiologist who was blinded to the histological diagnosis. To determine the inter-observer reproducibility, another neuroradiologist used the same method to delineate the ROIs in 30 randomly selected patients. Semi-automatic delineation was achieved by setting the optimal threshold range and proper combination of ROIs, including “AND”, “OR” and “XOR”. First, a proper threshold range of signal intensity was set for each subject to ensure that the red color covered the area of interest as completely as possible but did not cover the surrounding structures. Next, the wand tool was used to automatically delineate the connective pixels as a ROI. If the ROI created covered the entire area of interest, then the ROI delineation was finished; otherwise, for example, if it only covered a portion of the area, additional ROIs covering the residual portions were created using another threshold range to perform the combination of ROIs using “OR”. In certain cases, “AND” (for selecting a common area of two or more ROIs) and “XOR” (for selecting different area of two or more ROIs) were also applied to flexibly draw the ROIs, depending on the situation. Example delineations of the ROIs are shown in Figure 5. The ROIs over the solid enhancing tumor were delineated according to transverse contrast-enhanced T1-FLAIR, and the ROIs over the NAWM and non-enhancing tumor were delineated according to the transverse T2-FSE. Cystic components, necrosis, hemorrhage and calcification were avoided in the delineation of the solid region of the tumor [9]. The ROIs were copied from transverse contrast-enhanced T1-FLAIR or T2-FSE to all metric maps.

Average MK, Ka, Kr, MD, FA and ADC values were calculated for each ROI. The values in the solid region of the tumor were normalized to the corresponding values in the contralateral NAWM of each patient to eliminate whole-brain variations between individuals [15].

### Pathology and immunohistochemistry

To ensure a match of the location between histopathology and MR imaging, we first determined the area of the solid tumor preoperatively via MR images, and then requested the neurosurgeon to obtain the corresponding tissue during surgery for the further histopathological analysis. The nature and grade of the tumor were determined according to the 2007 WHO classification [1]. Immunohistochemical staining for Ki-67 was performed using the Envision method (Clone No. UMAB107, dilution 1:300). The tumor sections were reviewed and quantified based on the percentage of positive cells in the highest density of the stained areas; all cells with nuclear staining of any intensity were considered positive, and the Ki-67 LI values were defined as the percentage of positive cells among the total cells counted [8].

### Statistical analysis

The data were analyzed using SPSS software (Version 19.0.0, IBM, Armonk, NY) and MedCalc software (https://www.medcalc.org/, version 11.4.2.0). The inter-observer variability of measurements in 30 randomly selected patients was evaluated using the intra-class correlation coefficient. The independent-samples *t*-test was used to compare the differences in metrics in the solid region of the tumor and Ki-67 LI between HGGs and LGGs. One-way ANOVA was further employed to compare the differences among grade II, III and IV gliomas, and the Student-Newman-Keuls tests were used for multiple comparisons. The ROC curves were applied to evaluate the diagnostic efficiency of each metric in grading gliomas and to determine the optimal cut-off values. The *Z* test was applied to compare the differences in AUCs among MK, MD, ADC and FA. A stepwise multiple logistic regression analysis of MK, MD, ADC and FA was also performed to find the most significant metric for differentiating between each pair of glioma grades. The Pearson correlation analysis was used to evaluate the correlation between Ki-67 expression and each metric. A default alpha level of 0.05 was used for all tests, and all the tests were two-tailed.

## SUPPLEMENTARY FIGURES AND TABLES


