# Supplementary material for: Diffusion kurtosis imaging can efficiently assess the glioma grade and cellular proliferation
Source: Oncotarget. 2015 Nov 3;6(39):42380–93. doi: 10.18632/oncotarget.5675 (PMC4747234; doi:10.18632/oncotarget.5675)
Supplement: Supplementary file 1 [file oncotarget-06-42380-s001.pdf]

SUPPLEMENTARY FIGURES AND TABLES

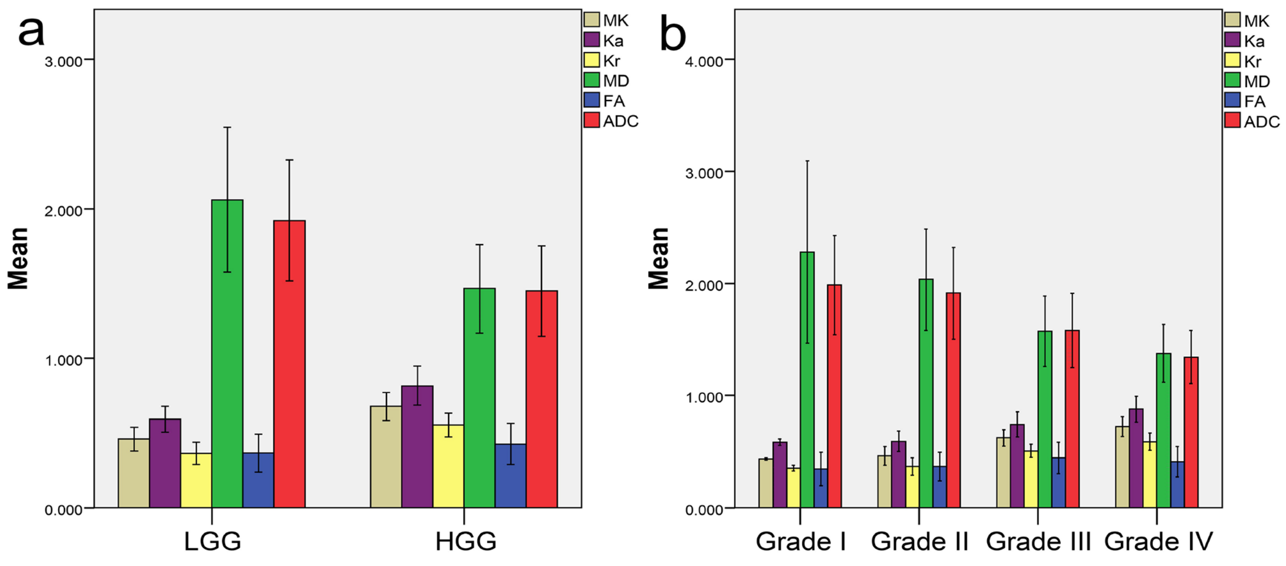

Supplementary Figure S1: Bar charts of normalized metrics for different grades of astrocytic tumors. Bar chart of each normalized metric **a.** in HGG and LGG and **b.** in grade I, grade II, grade III and grade IV astrocytic tumors. All metrics are dimensionless, except for MD and ADC, with units of 10^{-3} mm^2/sec.

MK: mean kurtosis; Ka: axial kurtosis; Kr: radial kurtosis; MD: mean diffusivity; FA: fractional anisotropy; ADC: apparent diffusion coefficient; HGG: high-grade glioma; LGG: low-grade glioma.

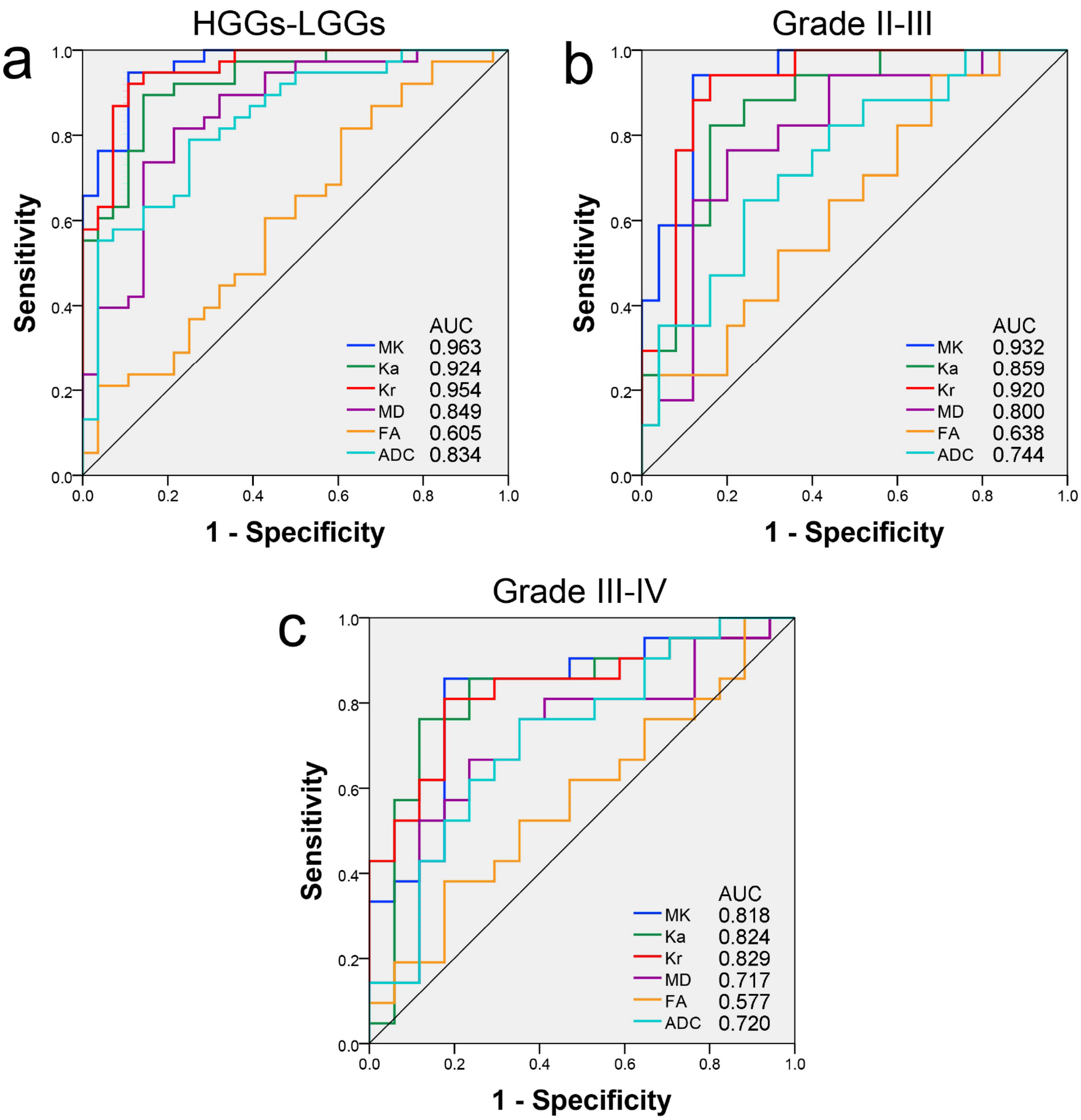

Supplementary Figure S2: ROC curves for all the metrics in differentiating different grades of astrocytic tumors. ROC curves and AUCs for all the metrics in the solid region of the tumor for differentiating **a.** between HGGs and LGGs, **b.** between grade II and III and **c.** between grade III and IV astrocytic tumors.

ROC curves: receiver operating characteristic curves; AUC: area under the curve; MK: mean kurtosis; Ka: axial kurtosis; Kr: radial kurtosis; MD: mean diffusivity; FA: fractional anisotropy; ADC: apparent diffusion coefficient; HGGs: high-grade gliomas; LGGs: low-grade gliomas.

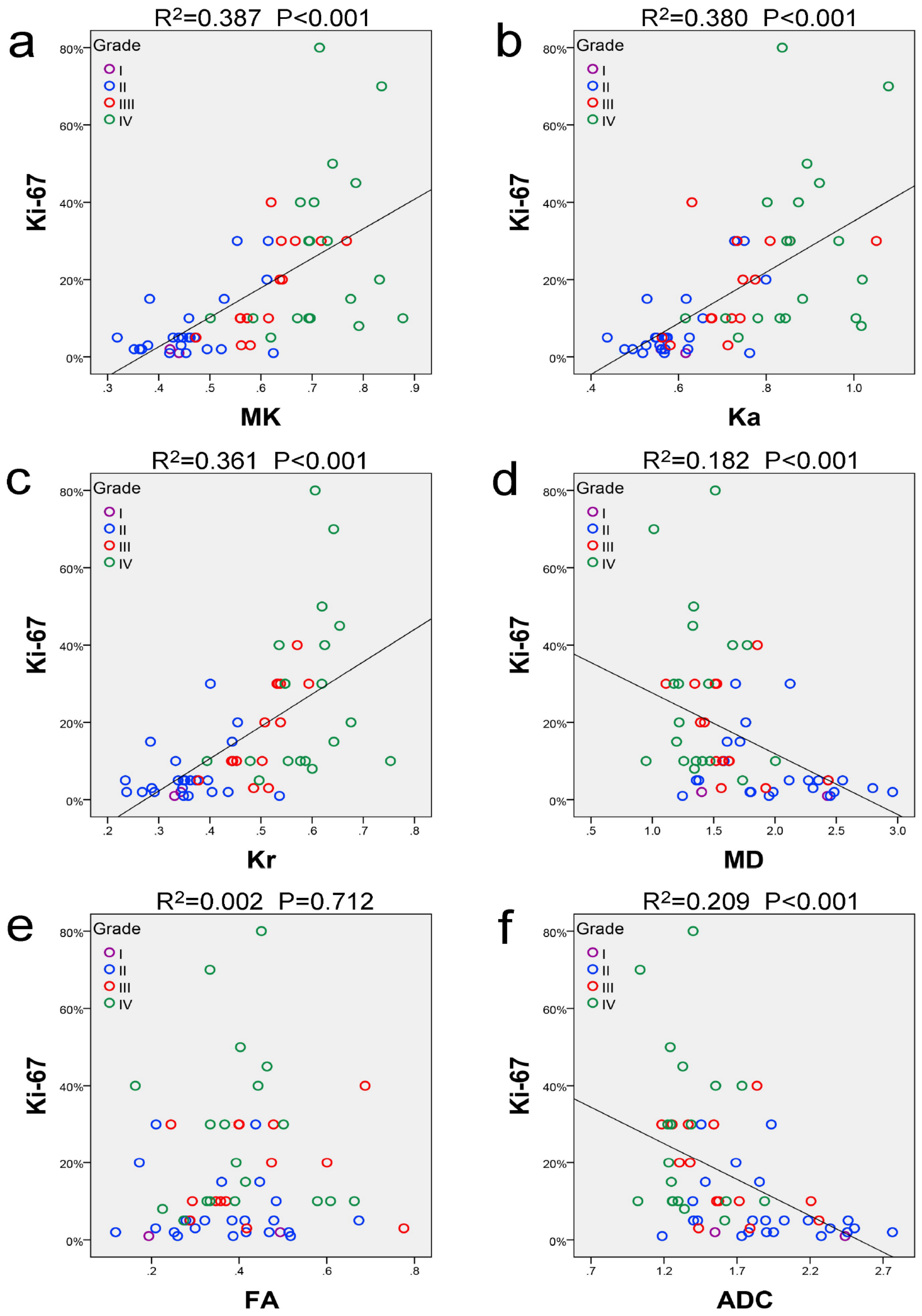

Supplementary Figure S3: Correlations between Ki-67 and each metric for astrocytic tumors. Scatter diagrams demonstrating the correlations between Ki-67 labeling index and **a.** MK, **b.** Ka, **c.** Kr, **d.** MD, **e.** FA or **f.** ADC. All metrics are dimensionless, except for MD and ADC, with units of 10^{-3} mm^2/sec.

MK: mean kurtosis; Ka: axial kurtosis; Kr: radial kurtosis; MD: mean diffusivity; FA: fractional anisotropy; ADC: apparent diffusion coefficient.

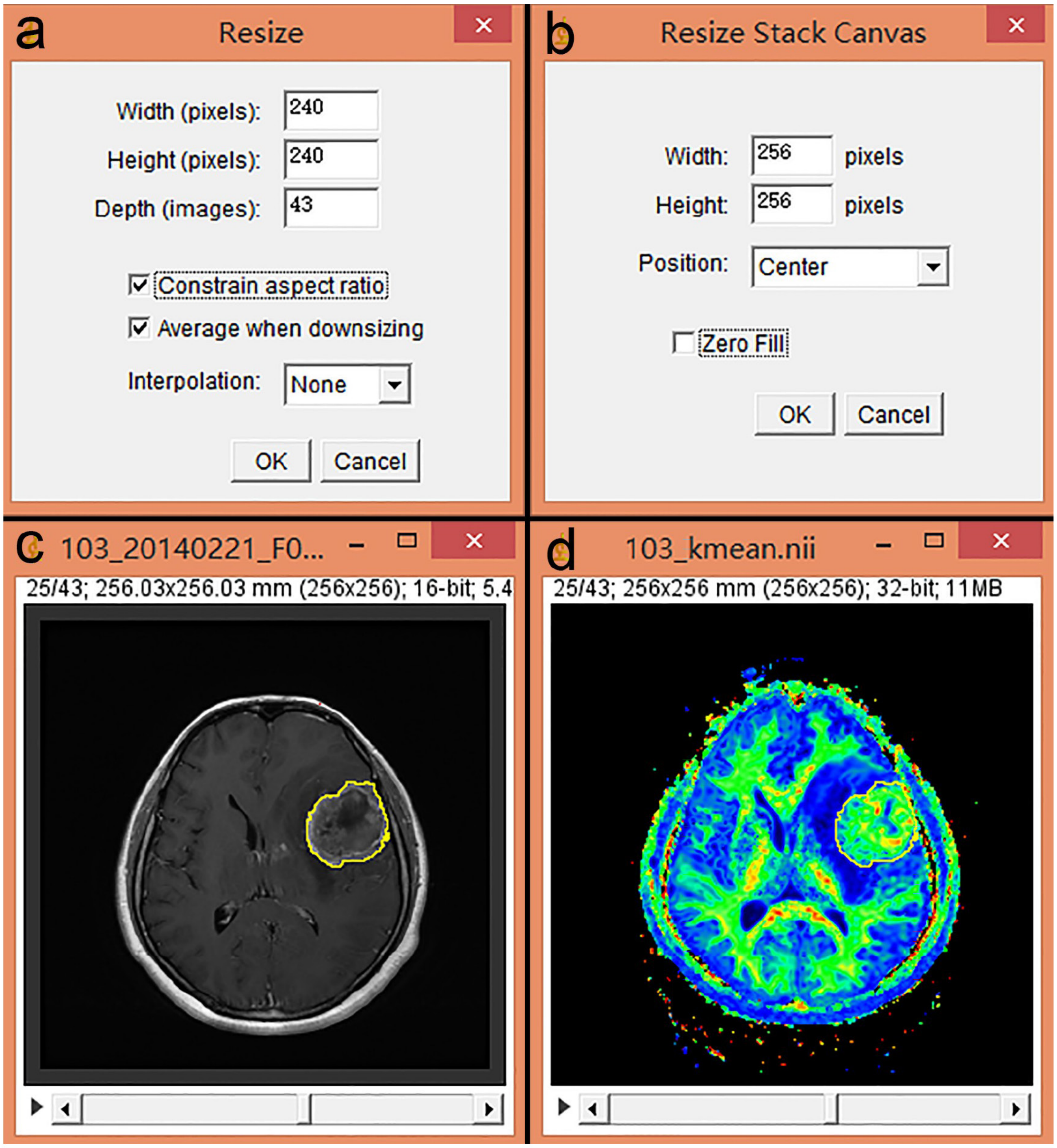

Supplementary Figure S4: Protocols for changing the resolution, number of slices and FOV of enhanced T1-FLAIR to match between enhanced T1-FLAIR and the DKI metric maps. The image resolutions ultimately generated by the scanner for enhanced T1-FLAIR and DKI was 512 × 512 and 256 × 256 due to interpolation. Thus, **a.** the image resolution of enhanced T1-FLAIR should first be resized to 240 × 240 (pixels were resized to 1mm × 1mm to match the DKI metric maps), and the number of slices should be changed to 43 without interpolation. **b.** The canvas size should then be adjusted to 256 × 256 due to the difference in FOV. **c.** ROI was semi-automatically delineated in enhanced T1-FLAIR, **d.** and finally copied to the DKI metric maps.

Supplementary Table S1: Detailed information for glioma patients

Category	Number	Age (years)
**Overall**	74	41.36 ± 14.01
**Gender**		
Male	44	41.68 ± 12.59
Female	30	40.90 ± 16.08
**Primary or recurrent**		
Primary glioma	67	40.93 ± 14.35
Recurrent glioma	7	45.57 ± 10.05
**Tumor excision or biopsy**		
Resection	74	41.36 ± 14.01
Biopsy	0	
**Grade**		
LGG	34	35.97 ± 15.17
HGG	40	45.95 ± 11.21
WHO grade I glioma	3	18.33 ± 15.31
Pilocytic astrocytoma	3	
WHO grade II glioma	31	37.68 ± 14.26
Diffuse astrocytoma	25	
Oligodendroglioma	3	
Oligo-astrocytoma	3	
WHO grade III glioma	19	43.53 ± 9.84
Anaplastic astrocytoma	16	
Gliomatosis cerebri	1	
Anaplastic oligodendroglioma	1	
Anaplastic oligo-astrocytoma	1	
WHO grade IV glioma	21	48.14 ± 12.14
Glioblastoma	21	

HGG: high-grade glioma; LGG: low-grade glioma

Supplementary Table S2: Descriptive statistics of tumor characteristics on routine MRI

Tumor characteristics	Overall	HGG	LGG	Grade I	Grade II	Grade III	Grade IV
**Size/area(cm^2)**							
In the maximal tumor slice	16.74 ± 9.81	17.96 ± 8.88	15.30 ± 10.76	20.93 ± 6.15	14.76 ± 11.02	19.58 ± 12.09	16.50 ± 4.19
In the solid region of the tumor	10.71 ± 7.57	10.72 ± 6.89	10.70 ± 8.41	7.91 ± 6.23	10.97 ± 8.62	10.06 ± 7.31	11.32 ± 6.61
**Intensity**							
Homogeneous	14	4	10	0	10	4	0
Not homogeneous	60	36	24	3	21	15	21
**Enhancement**							
Obvious enhancement	37	29	8	2	6	9	20
Mild enhancement	15	8	7	1	6	7	1
No enhancement	22	3	19	0	19	3	0
**Edge**							
Clear	39	12	27	3	24	6	6
Not clear	35	28	7	0	7	13	15
**Edema**							
Obvious edema	25	19	6	0	6	8	11
Mild edema	28	16	12	0	12	7	9
No edema	21	5	16	3	13	4	1
**Legion number**							
Multiple	16	13	3	0	3	4	9
Single	58	27	31	3	28	15	12

HGG: high-grade glioma; LGG: low-grade glioma

Supplementary Table S3: Ki-67 expression in gliomas of different grades

Grade	Number	Ki-67(%)
Overall	66	15.091 ± 16.527
LGGs	31	6.677 ± 7.735
HGGs	35	22.543 ± 18.646
Grade I gliomas	2	1.500 ± 0.707
Grade II gliomas	29	7.035 ± 7.876
Grade III gliomas	16	16.625 ± 11.960
Grade IV gliomas	19	27.526 ± 21.912

Note: Data are presented as the mean and standard deviation.
LGGs: low-grade gliomas; HGGs: high-grade gliomas.

Supplementary Table S4: Comparisons of the kurtosis and conventional diffusion metrics in the grading of astrocytic tumors

Grade	*N*	MK	Ka	Kr	MD	FA	ADC
Total	66						
LGG	28	0.458 ± 0.078	0.590 ± 0.085	0.363 ± 0.074	2.060 ± 0.487	0.364 ± 0.127	1.921 ± 0.405
HGG	38	0.676 ± 0.095	0.816 ± 0.132	0.552 ± 0.080	1.465 ± 0.297	0.424 ± 0.137	1.450 ± 0.303
P		< 0.001*	< 0.001*	< 0.001*	< 0.001*	0.075	< 0.001*
Grade II	25	0.460 ± 0.082	0.591 ± 0.090	0.365 ± 0.078	2.034 ± 0.452	0.367 ± 0.127	1.913 ± 0.410
Grade III	17	0.621 ± 0.072	0.741 ± 0.112	0.507 ± 0.059	1.575 ± 0.312	0.444 ± 0.141	1.582 ± 0.331
Grade IV	21	0.721 ± 0.089	0.877 ± 0.115	0.588 ± 0.077	1.376 ± 0.259	0.409 ± 0.136	1.344 ± 0.236
P		< 0.001*	< 0.001*	< 0.001*	< 0.001*	0.187	< 0.001*
SNK		IV > III > II	IV > III > II	IV > III > II	II > III, IV	III, IV, II	II > III > IV

Note: All metrics are dimensionless, except for MD and ADC, with units of 10^{-3} mm^2/sec.

$^*P < 0.05$.

In the results of SNK, "A > B" indicates that the metric value of A is significantly higher than that of B; "A, B" indicates that the metric value of A is higher than that of B, but without reaching significance.

HGG: high-grade glioma; LGG: low-grade glioma; MK: mean kurtosis; Ka: axial kurtosis; Kr: radial kurtosis; MD: mean diffusivity; FA: fractional anisotropy; ADC: apparent diffusion coefficient; SNK: Student-Newman-Keuls.

Supplementary Table S5: Statistical values of all metrics for differentiating between HGGs and LGGs, grade II and grade III, and grade III and grade IV astrocytic tumors

Metrics	AUC	*P* Value	Cut-off value	Sensitivity	Specificity
**HGGs-LGGs**					
MK	0.963	< 0.001*	0.553	94.70%	89.30%
Ka	0.924	< 0.001*	0.655	89.50%	85.70%
Kr	0.954	< 0.001*	0.443	92.10%	89.30%
MD(10^{-3} mm^2/sec)	0.849	< 0.001*	1.655	81.60%	78.60%
FA	0.605	0.142	0.322	81.60%	39.30%
ADC(10^{-3} mm^2/sec)	0.834	< 0.001*	1.627	78.90%	75.00%
**Grade II–III**					
MK	0.932	< 0.001*	0.553	94.10%	88.00%
Ka	0.859	< 0.001*	0.655	82.40%	84.00%
Kr	0.920	< 0.001*	0.436	94.10%	84.00%
MD(10^{-3} mm^2/sec)	0.800	< 0.001*	1.627	76.50%	80.00%
FA	0.638	0.115	0.287	94.10%	32.00%
ADC(10^{-3} mm^2/sec)	0.744	0.002	1.575	64.70%	76.00%
**Grade III–IV**					
MK	0.818	< 0.001*	0.667	85.70%	82.40%
Ka	0.824	< 0.001*	0.825	76.20%	88.20%
Kr	0.829	< 0.001*	0.538	81.00%	82.40%
MD(10^{-3} mm^2/sec)	0.717	0.013*	1.409	66.70%	76.50%
FA	0.577	0.418	0.334	38.10%	82.40%
ADC(10^{-3} mm^2/sec)	0.720	0.010*	1.400	76.20%	64.70%

Note: The cut-off value indicates the optimal threshold in the current study/sample size. All metrics are dimensionless, except for MD and ADC.

$^{*}P < 0.05$.

AUC: area under the curve; HGGs: high-grade gliomas; LGGs: low-grade gliomas; MK: mean kurtosis; Ka: axial kurtosis; Kr: radial kurtosis; MD: mean diffusivity; FA: fractional anisotropy; ADC: apparent diffusion coefficient.

Supplementary Table S6: Comparisons of AUCs among MK, MD and FA in astrocytic tumors

Comparison	Statistic	MK-MD	MK-ADC	MK-FA	MD-ADC	MD-FA	ADC-FA
LGG-HGG	P	0.008*	0.001*	< 0.001*	0.564	< 0.001*	< 0.001*
	Z	2.675	3.262	5.031	0.577	3.685	3.410
Grades II–III	P	0.029*	0.002*	< 0.001*	0.191	0.097	0.257
	Z	2.185	3.036	3.399	1.308	1.662	1.135
Grades III–IV	P	0.189	0.187	0.053	0.940	0.293	0.278
	Z	1.314	1.320	1.934	0.075	1.052	1.085

Note:

$^{*}P < 0.05$.

MK: mean kurtosis; MD: mean diffusivity; ADC: apparent diffusion coefficient; FA: fractional anisotropy; HGGs: high-grade gliomas; LGGs: low-grade gliomas.

Supplementary Table S7: Stepwise multiple logistic regression analyses of metrics in the prediction of astrocytic tumor grades

Differentiation	Variable	Coefficient	OR (95% CI)	*P*	Variables not included in the model
HGG-LGG	MK	2.671	14.455 (4.106–50.885)	< 0.001	MD, ADC, FA
Grades II–III	MK	2.539	12.667 (2.919–54.970)	< 0.001	MD, ADC, FA
Grades III–IV	MK	1.542	4.674 (1.604–13.615)	< 0.001	MD, ADC, FA

Note: The coefficient, OR, 95% CI and P indicate the corresponding values when MK increases by 0.1.
OR: odd ratio; CI: confidence interval; MK: mean kurtosis; MD: mean diffusivity; ADC: apparent diffusion coefficient; FA: fractional anisotropy; HGG: high-grade glioma; LGG: low-grade glioma.

Supplementary Table S8: Correlations between Ki-67 and each metric for astrocytic tumors

		MK	Ka	Kr	MD	FA	ADC
Ki-67	*r*	0.622	0.617	0.601	–0.427	0.050	–0.457
	P	< 0.001*	< 0.001*	< 0.001*	< 0.001*	0.712	< 0.001*
	N	58	58	58	58	58	58

Note:
$^{*}P < 0.05$.
MK: mean kurtosis; Ka: axial kurtosis; Kr: radial kurtosis; MD: mean diffusivity; FA: fractional anisotropy; ADC: apparent diffusion coefficient.
